# Relaparotomy Two Years after Incisional Hernia Repair Using a Free Fascia Lata Graft

**DOI:** 10.1155/2020/1769404

**Published:** 2020-03-13

**Authors:** Yuuki Sekine, Hiroyuki Sugo, Naoki Iwanaga, Shigefumi Neshime, Ikuo Watanobe

**Affiliations:** Department of General Surgery, Juntendo University Nerima Hospital, 3-1-10 Takanodai, Nerima-ku, Tokyo 177-8521, Japan

## Abstract

Although, free fascia lata autografts can be used to reconstruct various anatomical structures, little information is available about the status of such autografts several years after the procedure, especially in a clinical setting. Here, we describe our experience with a patient who underwent relaparotomy two years after incisional hernia repair using a fascia lata graft. A 79-year-old man underwent open hepatectomy for hepatocellular carcinoma. One year later, abdominal computed tomography revealed a locally recurrent tumor 1.5 cm in diameter and a giant incisional hernia measuring approximately 15 × 6 cm on the supraumbilical midline. After repeat hepatectomy, the incisional hernia was repaired using a free fascia lata patch as an interpositional graft. Two years later, the patient was readmitted because of recurrent tumors in the liver, and repeat hepatectomy was performed. During surgery, the fascia lata graft had survived well and become incorporated into the native fascia. We incised this fascia lata graft in the same way as for a normal laparotomy. After hepatectomy, the fascia lata graft was closed in layers with interrupted sutures. The patient was discharged on postoperative day 11 with no wound-related morbidity.

## 1. Introduction

Reconstruction of the abdominal wall and incisional hernia repair have been performed using an autologous tissue graft as an alternative to prosthetic materials, especially in patients with a risk of surgical site infection [1–3]. A free fascia lata graft is used to reconstruct various anatomical structures as an autologous tissue graft. Despite the large number of patients who have undergone reconstructive surgery using fascia lata autografts, little information is available on the status of such autografts several years after the procedure.

Here, we describe our experience with a patient who underwent relaparotomy two years after incisional hernia repair using a fascia lata graft. To our knowledge, no previous reports have described details of relaparotomy in patients with autologous tissue grafts.

## 2. Case Presentation

**Figure 1:**
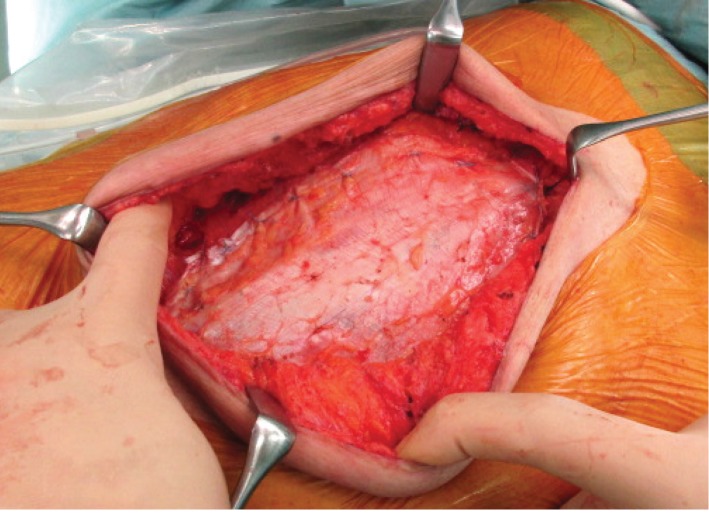
Intraoperative view of the hernia repair two years previously: the fascia lata graft patch was placed as an interpositional graft covering the rectus abdominis muscle defect.

A 79-year-old man underwent open hepatectomy for a hepatocellular carcinoma 4.3 cm in diameter. One year later, abdominal computed tomography revealed a locally recurrent tumor 1.5 cm in diameter and a giant incisional hernia measuring approximately 15 × 6 cm on the supraumbilical midline. Consequently, the incisional hernia was repaired using a free fascia lata graft patch after repeat hepatectomy. During the surgery, the fascia lata was harvested from the left thigh. An incision approximately 12 cm long was made on the lateral side of the thigh to allow for the dissection of subcutaneous tissue and harvest a piece of fascia lata measuring 16 × 8 cm. After the hepatectomy, the skin and subcutaneous tissues were dissected down to the level of the anterior rectus sheath on both sides of the defect, exposing the native fascia along the edge of the defect. After suturing 2 cm of the top and bottom edges of the native fascia lata, the fascia lata graft was used to reconstruct the remaining defect, which measured 12 × 6 cm. For placement of the graft, the fascia lata was sutured superficial to the edge of the defect with minimal overlap (interpositional graft) (Figure 1) [4].

**Figure 2:**
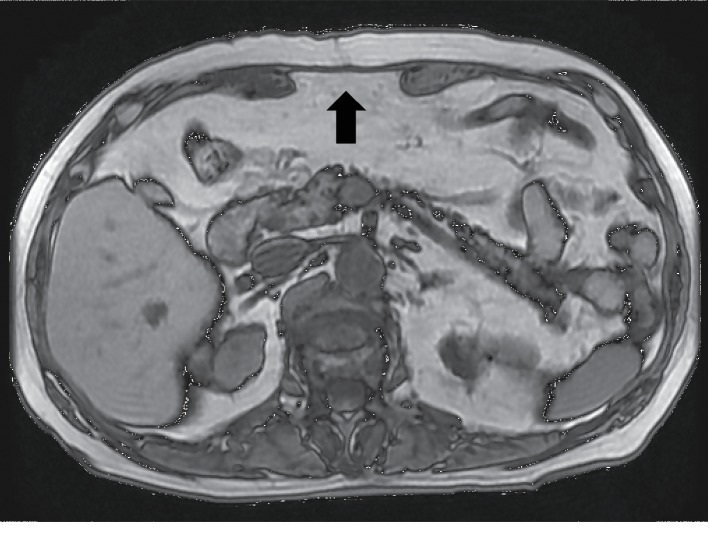
Magnetic resonance imaging reveals the implanted fascia lata graft (arrow).

**Figure 3:**
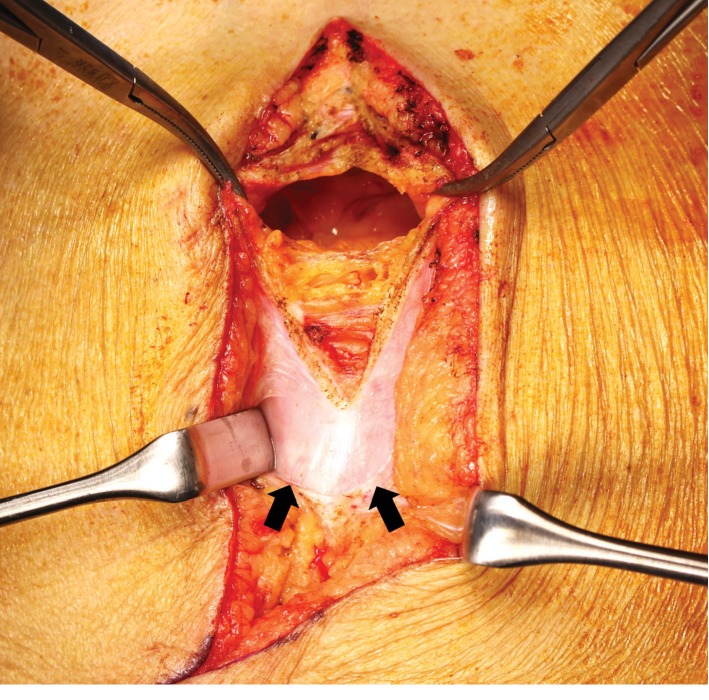
Intraoperative view: the implanted fascia below the subcutaneous tissue (arrows).

**Figure 4:**
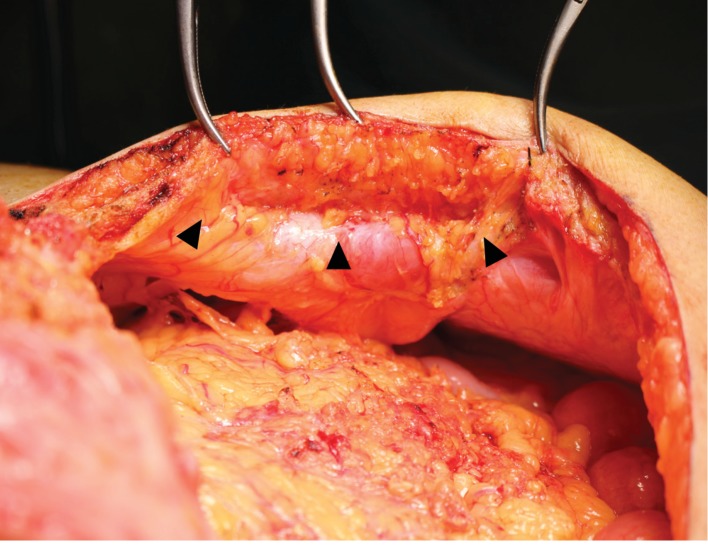
Reverse side view of the implanted fascia lata graft: both the cut edge of the implanted fascia graft (arrowheads) and the edges of the rectus abdominis muscle (dotted line) lie along the incision line.

Two years later, the patient was readmitted because of recurrent tumors measuring 3.0 and 1.0 cm in the S5 and S7 subsegments of the liver, respectively. Preoperative magnetic resonance imaging (MRI) revealed the implanted fascia lata graft above the sheath of the rectus abdominis as an interpositional graft (Figure 2). Repeat hepatectomy was performed. A ventral midline incision was made and the skin and subcutaneous tissues were dissected down to the level of the fascia, which was white and hard, similar to an implanted fascia lata graft (Figure 3). The fascia lata graft had survived well and become incorporated into the native fascia. We incised this fascia lata graft in the same way as for a normal laparotomy (Figure 4). After limited hepatectomy had been performed, the midline abdominal fascia, including the fascia lata graft and the skin, was closed in layers with interrupted suture and skin staples, respectively. The patient was discharged on postoperative day 11 with no wound-related morbidity or hernia recurrence. Three months after surgery, MRI revealed that the resutured fascial graft had healed completely to the same state as that evident preoperatively, and the patient is doing well for 11 months after surgery without hernia recurrence.

## 3. Discussion

Although prosthetic materials are commonly used to repair abdominal fascial defects, autologous tissue is preferred in the presence of contamination due to the risk of mesh infection. In such a situation, autologous tissue grafts have better adaptability and a lower risk of infection in comparison with prosthetic materials [4, 5]. The Ventral Hernia Working Group (VHWG) recommends that biological repair materials are preferable to synthetic mesh for use in infected fields and should be strongly considered when contamination is suspected [4]. In the present case, we selected an autologous fascia graft for incisional hernia repair because of the risk of contamination due to concomitant hepatectomy.

Regarding the viability of fascia autografts, Peacock reported that none of the 17 patients who underwent the onlay imbrication technique suffered recurrences over an observation period of 2-5 years and that there were no indications of graft disintegration [6]. Other clinical studies also demonstrated that fascia grafts remain viable after implantation [2]. Moreover, several experimental studies have confirmed that the grafts become revascularized [2, 7]. On the other hand, the status of fascia autografts several years after the procedure has been unclear from a clinical viewpoint. No previous reports have given details of relaparotomy for patients who have undergone reconstructive surgery using fascia lata autografts, especially in a clinical setting. This is the first clinical report to have given details of relaparotomy in a patient with autologous tissue grafts. In the present case, the implanted fascia was suggested to be revascularized from preoperative MRI findings and without hernia recurrence, then we indicated a ventral midline incision including the incision of fascia lata graft. Consequently, the implanted fascia had survived well and become incorporated into the native fascia at the time of relaparotomy. We then closed the incised fascia again in layers with interrupted absorbable sutures in the same bite and interval as for a normal. Postoperatively, the resutured fascia healed well, as revealed by postoperative MRI.

Wound and mesh infections can be a grave complication in patients undergoing repair with prosthetic materials [3]. If mesh infection occurs, it is very difficult to resolve, and the situation usually requires complete removal of the mesh and sometimes multiple surgical interventions. On the other hand, Disa et al. demonstrated that fascial grafts became revascularized and incorporated as living tissue and were more resistant to bacterial contamination than prosthetic patches in an experimental animal study [2]. Although repeat hepatectomy was required in this patient, no wound-related morbidity including infection was observed despite the risk of contamination. From this viewpoint, relaparotomy is more advantageous for patients with fascial graft repair than for those with prosthetic material repair.

In conclusion, the present case suggests that the status of an implanted fascia graft is the same as that of native fascia in terms of healing and resistance to contamination. Further studies are required to evaluate the long-term status of implanted fascial grafts. In our limited experience, we suggest that relaparotomy is safe after repair with a free fascia lata graft.
